# Improved Consistency in Dosing Anti-Tuberculosis Drugs in Taipei, Taiwan

**DOI:** 10.1371/journal.pone.0044133

**Published:** 2012-08-27

**Authors:** Chen-Yuan Chiang, Ming-Chih Yu, Hsiu-Chen Shih, Muh-Yong Yen, Yu-Ling Hsu, Shiang-Lin Yang, Tao-Ping Lin, Kuan-Jen Bai

**Affiliations:** 1 International Union Against Tuberculosis and Lung Disease, Paris, France; 2 Division of Pulmonary Medicine, Department of Internal Medicine, Wan Fang Hospital, Taipei Medical University, Taipei, Taiwan; 3 Department of Internal Medicine, School of Medicine, College of Medicine, Taipei Medical University, Taipei, Taiwan; 4 Department of Disease Control and Prevention, Taipei City Hospital, Taipei City Government, Taipei, Taiwan; 5 Centers for Disease Control, Department of Health, Taipei, Taiwan; 6 Taiwan Anti-Tuberculosis Association, Taipei, Taiwan; Barcelona University Hospital, Spain

## Abstract

**Background:**

It was reported that 35.5% of tuberculosis (TB) cases reported in 2003 in Taipei City had no recorded pre-treatment body weight and that among those who had, inconsistent dosing of anti-TB drugs was frequent. Taiwan Centers for Disease Control (CDC) have taken actions to strengthen dosing of anti-TB drugs among general practitioners. Prescribing practices of anti-TB drugs in Taipei City in 2007–2010 were investigated to assess whether interventions on dosing were effective.

**Methodology/Principal Findings:**

Lists of all notified culture positive TB cases in 2007–2010 were obtained from National TB Registry at Taiwan CDC. A medical audit of TB case management files was performed to collect pretreatment body weight and regimens prescribed at commencement of treatment. Dosages prescribed were compared with dosages recommended. The proportion of patients with recorded pre-treatment body weight was 64.5% in 2003, which increased to 96.5% in 2007–2010 (p<0.001). The proportion of patients treated with consistent dosing of a 3-drug fixed-dose combination (FDC) increased from 73.9% in 2003 to 87.7% in 2007–2010 (p<0.001), and that for 2-drug FDC from 76.0% to 86.1% (p = 0.024), for rifampicin (RMP) from 62.8% to 85.5% (p<0.001), and for isoniazid from 87.8% to 95.3% (p<0.001). In 2007–2010, among 2917 patients treated with either FDCs or RMP in single-drug preparation, the dosage of RMP was adequate (8–12 mg/kg) in 2571(88.1%) patients, too high in 282(9.7%), too low in 64(2.2%). In multinomial logistic regression models, factors significantly associated with adequate dosage of RMP were body weight and preparations of RMP. Patients weighting <40kg (relative risk ratio (rrr) 6010.5, 95% CI 781.1–46249.7) and patients weighting 40–49 kg (rrr 1495.3, 95% CI 200.6–11144.6) were more likely to receive higher-than-recommended dose of RMP.

**Conclusions/Significance:**

Prescribing practice in the treatment of TB in Taipei City has remarkably improved after health authorities implemented a series of interventions.

## Introduction

Tuberculosis (TB) services were provided by general practitioners in Taiwan. TB was a notifiable disease by law. Completeness of TB notification in Taiwan has been investigated; 96.3% of patients who were prescribed 2 or more anti-TB drugs in 2005–2007 were notified to Taiwan Centers for Disease Control (CDC) [1]. Prescription of adequate dosage of anti-tuberculosis drugs is essential in the treatment of TB [2], [3]. Chiang and colleagues assessed general practitioners' prescribing practice of anti-TB medicines in Taiwan and reported that 35.5% of TB cases reported in the year 2003 in Taipei City had no recorded pre-treatment body weight and that among those who had, inconsistent dosing of anti-TB drugs was frequent [4]. Of 506 patients prescribed a three-drug fixed-dose combination (FDC), the dosage was consistent with guidelines in 374 (73.9%), too low in 100 (19.8%) and too high in 32 (6.3%). Of 481 patients prescribed rifampicin (R, RMP), the dosage was consistent with guidelines in 302 (62.8%), too low in 152 (31.6%) and too high in 27 (5.6%). Of 451 patients prescribed isoniazid (H, INH), the dosage was consistent with guidelines in 396 (87.8%), too low in 29 (6.4%) and too high in 26 (5.8%).

Prescription of anti-TB drugs within national TB programme (NTP) routinely follows NTP manual and is usually more standardized than that among general practitioners. Studies conducted under programme conditions reported that the proportions of patients with recorded pre-treatment body weight were 73% in Kenya, 82% in Nepal, 98% in Senegal and 98% in Malawi [5], [6]. Among those with recorded pretreatment body weight, the proportion of patients prescribed adequate dosages of INH was 35% in Malawi, 33.7% in Kenya, 14.7% in Nepal and 14.5% in Senegal, and the proportion of patients prescribed adequate dosage of RMP was 96% in Malawi, 76.5% in Kenya, 77.3% in Nepal and 92.9% in Senegal.

Review of prescribing practices has been indicated as an essential tool for measuring the quality of TB services [7]. The question is whether inconsistent dosing of anti-TB drugs among general practitioners can be corrected by proper interventions. Findings of inconsistent dosing of anti-TB drugs in Taipei were reported to Taiwan CDC in December 2005. Thereafter, Taiwan CDC and health authorities have taken actions to address inconsistent dosing of anti-TB drugs. Collaboration between Taiwan CDC and professional societies (such as Taiwan Society of Tuberculosis, Taiwan Society of Pulmonary and Critical Care Medicine) was strengthened. Guidelines for diagnosis and treatment of TB were updated. Several TB training courses targeting general practitioners were held. Those who completed training were qualified as TB services providers. Those who had comprehensive training and passed an examination organized by Taiwan Society of Tuberculosis were certified as TB specialists. TB expert committees were organized to provide guidance in the diagnosis and treatment of TB. In 2006, TB treatment cards were revised to capture pretreatment body weight. In the past Taiwan CDC restricted its activities to public health function and did not intervene in clinical practice. This approach was changed in 2007 when public health nurses were trained to assess prescribing practices in the treatment of TB. In case of inconsistent dosing, public health nurses would contact prescribing clinicians, and if inconsistency remains after initial contact, would report such cases to TB expert committees for further elaboration. TB expert committees held meetings periodically to review TB cases whose diagnosis or treatment was judged to be problematic by public health nurses. Recommendations of TB expert committees were communicated to general practitioners who must respond (agree or disagree) to expert committee's recommendation. In 2008, Taiwan CDC collaborated with the national health insurance (NHI) program to promote standardized anti-TB regimens; reimbursement was drastically reduced in case of inconsistent dosing of anti-TB drugs [8], [9].

**Figure 1:**
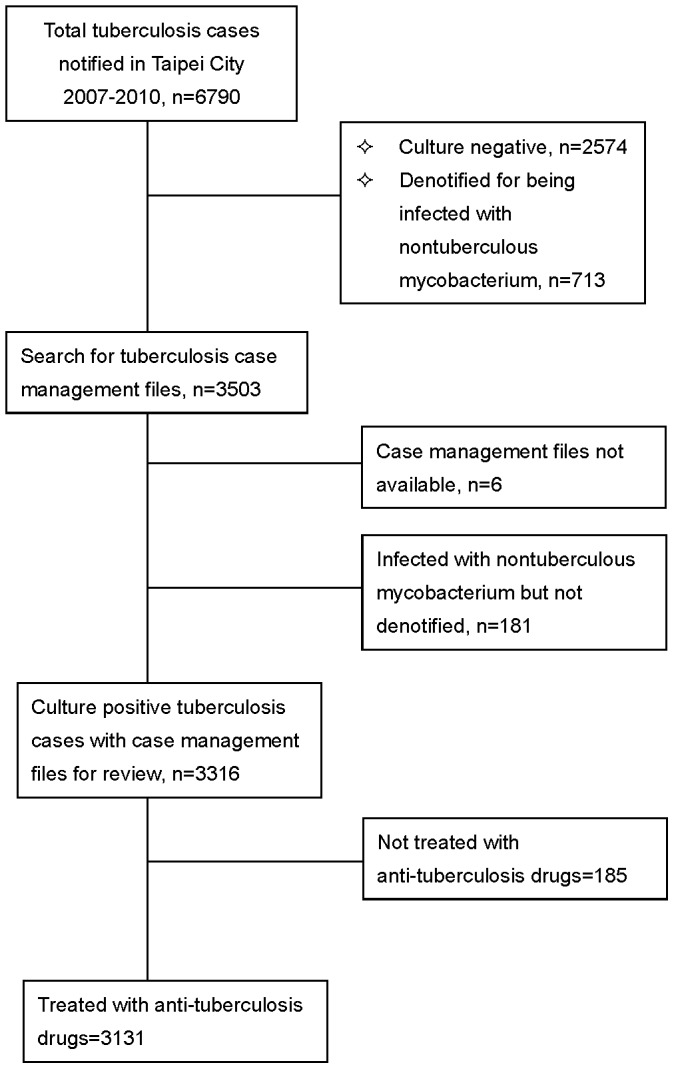
Flow chart of study population.

**Table 1:** Characteristics of 2990 adults with pre-treatment body weight.

Characteristics	Number (%)
Sex	
Male	2059 (68.9%)
Female	931(31.1%)
Age group (years old)	
≤24	148 (5.0%)
25–44	473(15.8%)
45–64	713(23.9%)
≥65	1656(55.4%)
History of tuberculosis	
New	2678(89.6%)
Previously treated	312(10.4%)
Pretreatment body weight (kilogram)	
≤29	7(0.2%)
30–39	139(4.7%)
40–49	808(27.0%)
≥50kg	2036(68.1%)
Diabetes Mellitus	
Yes	545(18.2%)
No	2063(69.0%)
Unknown	382(12.8%)
Chronic liver diseases	
Yes	122(4.1%)
No	2486(83.1%)
Unknown	382(12.8%)
Chronic renal failure	
Yes	64(2.1%)
No	2544(85.1%)
Unknown	382(12.8%)

To assess whether these interventions on dosing were effective, a clinical audit was done to investigate prescribing practices of anti-TB drugs in Taipei City in 2007–2010. Primary objective was to assess whether there was difference between 2003 and 2007–2010 in prescribing practice of anti-TB drugs in Taipei City. Secondary objective was to investigate factors associated with inconsistent dosing of anti-TB drugs. We report results of this investigation.

**Figure 2:**
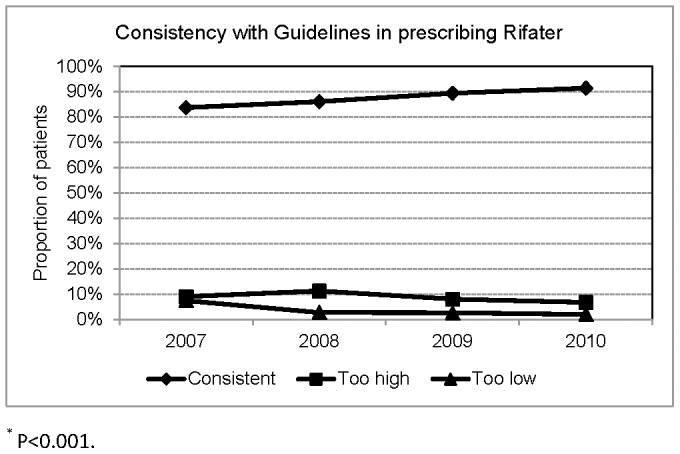
Consistency with Taiwan Guidelines in prescribing Rifater.

## Materials and Methods

### Research questions

We tested the null hypothesis that interventions addressing inconsistent dosing were not effective and that there was no difference between 2003 and 2007–2010 in terms of the proportion of patients with recorded pretreatment body weight and the proportions of patients with consistent dosing of 3-drug FDC, 2-drug FDC, RMP, and INH. We extended the audit to ethambutol (E, EMB) and pyrazinamide (Z, PZA) as well.

**Figure 3:**
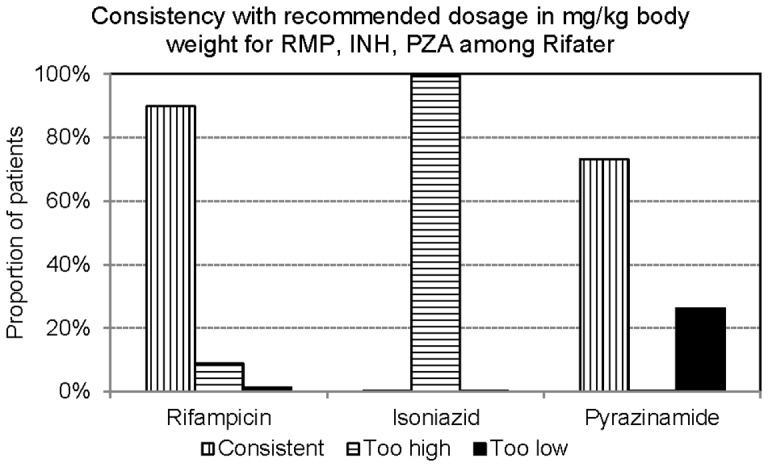
Consistency with recommended dosage in mg/kg body weight for Rifampicin 8–10 mg/kg), Isoniazid (4–6 mg/kg), and pyrazinamide (20–30 mg/kg) among those prescribed Rifater.

**Figure 4:**
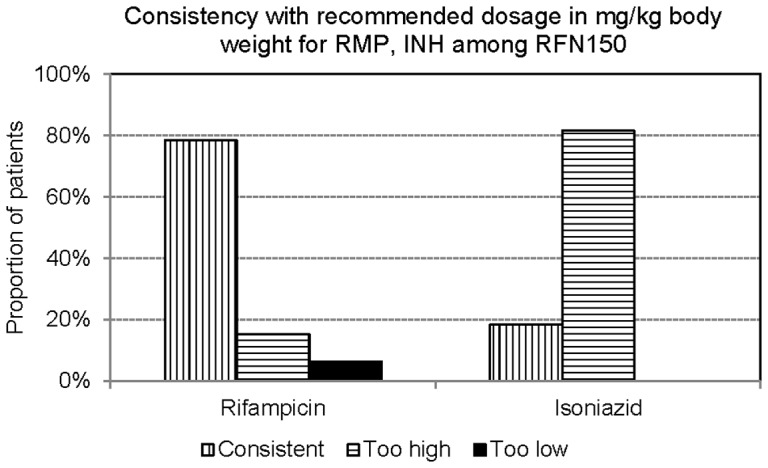
Consistency with recommended dosage in mg/kg body weight for Rifampicin 8–10 mg/kg) and Isoniazid (4–6 mg/kg) among those prescribed Rifinah150.

### Study population

The study population included all individuals with culture positive for *Mycobacterium tuberculosis* who were citizens of Taipei City reported to Taiwan CDC in the calendar year 2007–2010. Lists of all notified TB cases of Taipei City in 2007–2010 were obtained from National TB Registry at Taiwan CDC. Each TB case had a case management file maintained by public health nurses at Department of Disease Control and Prevention, Taipei City Department of Health. It contained information on history of TB, sputum examinations (smear, culture, identification, and susceptibility testing), body weight, prescription of anti-TB drugs (type of drugs and dosage), outcome of treatment, as well as age, sex, symptom profiles, family history of TB, concomitant diseases (diabetes, liver diseases, renal disease, gastrectomy, cancers, use of steroids, HIV, etc), smoking, use of alcohol beverage, and contact examinations. A structured questionnaire was designed for this audit and a team of 11 individuals were organized; the majority of them were senior nurses with substantial experience of TB services. The questionnaire was discussed, pilot tested, and revised for 4 times before it was finalized for data collection. A medical audit of TB case management files was performed in 2011 to collect pretreatment body weight and regimens prescribed at commencement of treatment. Dosages prescribed were compared with dosages recommended by Taiwan Guidelines for the diagnosis and treatment of TB [10]. Modification of regimens after initial prescription was not assessed. Each completed questionnaire was double checked by another team member to ensure accuracy and consistency of data collection.

**Figure 5:**
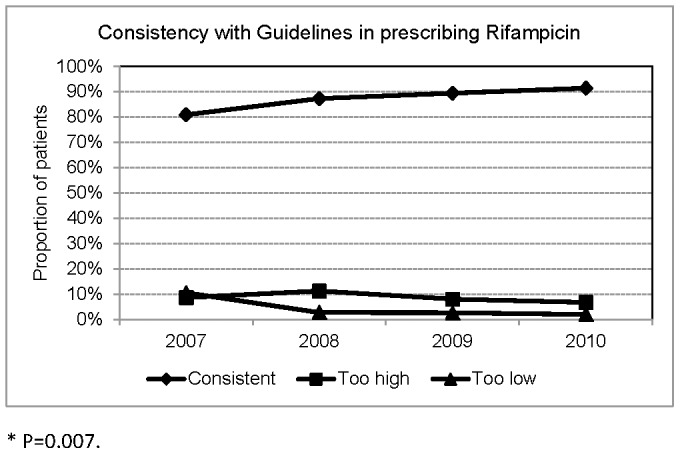
Consistency with Taiwan Guidelines in prescribing Rifampicin.

### Dosing of fixed-dose combinations

Two types of FDC of anti-TB drugs were available in Taiwan, one containing three and the other two drugs. The three-drug tablet (Rifater) contains INH 80 mg, RMP 120 mg and PZA 250 mg (the ratios of H:R:Z were 1∶1.5∶3.1). The two-drug tablet was available in two different forms, one (Rifinah150, RFN150) containing INH 100 mg and RMP 150 mg (the ratio of H:R was 1∶1.5), and the other (Rifinah300, RFN300) with INH 150 mg and RMP 300 mg (the ratio of H:R was 1∶2). Concerning the 3-drug FDC, Taiwan CDC recommended 3 tablets per day for adults weighing <40 kg, 4 tablets for 40–49 kg and 5 tablets for ≥50 kg; and concerning the 2-drug FDC, 3 tablets of RFN150 (total H 300 mg+R 450 mg) for adults weighing <50 kg and 2 tablets of RFN300 per day (total H 300 mg+R 600 mg) for adults weighing ≥50 kg.

**Figure 6:**
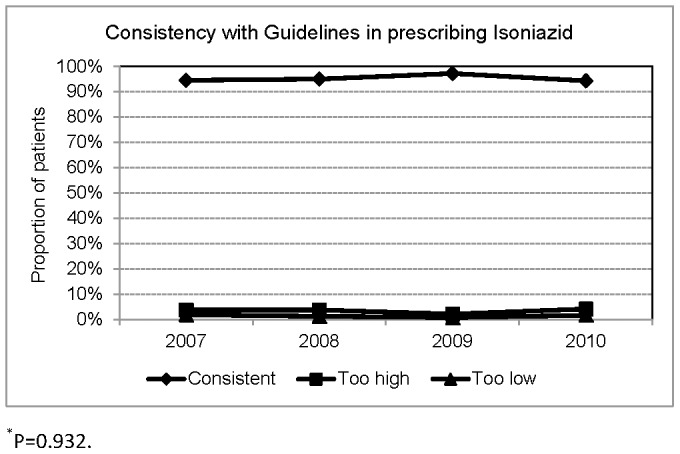
Consistency with Taiwan Guidelines in prescribing Isoniazid.

### Dosing of RMP in single-drug preparation

Taiwan CDC recommended 600 mg RMP for patients weighing ≥50 kg and 450 mg for those weighing <50 kg. The alternative recommendation is to prescribe 10 milligrams per kilograms (mg/kg) body weight (range 8–12 mg/kg) with 600 mg as the maximal dose.

**Figure 7:**
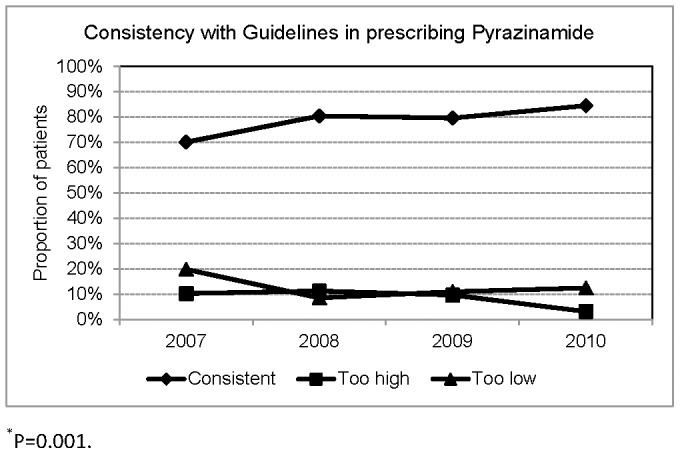
Consistency with Taiwan Guidelines in prescribing Pyrazinamide.

### Dosing of INH in single-drug preparation

Taiwan CDC recommended 300 mg INH for the majority of patients and 200 mg for those with low body weight. Following the previous study, [4] patients weighing <40 kg were defined as patients with low body weight. The alternative recommendation is to prescribe 5 (range 4–6) mg/kg body weight with 300 mg as the maximal dose.

**Table 2:** Factors associated with inadequate dosing of rifampin among patients treated with Rifater, Rifinah, or Rifampicin in single-drug preparation, 2007–2010, Taipei.

	Adequate	Too high		Too low	
	Number (%)	Relative RR	95% CI	Relative RR	95% CI
Weight (kg)					
≤39	63(45.0%)	1		1	
40–49	576(72.8%)	0.25	0.17–0.37	0.30	0.08–1.07
≥50	1932(97.3%)	0.0002	0.00002–0.001	1.19	0.38–3.72
Preparations					
Rifampicin	459(83.0%)	1		1	
Rifater	1663(89.9%)	0.99	0.69–1.41	0.21	0.12–0.37
Rifinah150	102(77.9%)	0.46	0.25–0.85	2.21	0.97–5.01
Rifinah300	347(90.4%)	8.85	4.29–18.25	0.04	0.005–0.27

Adequate dosage (8–12 mg/kg) as the base for comparison. Too high, >12 mg/kg. Too low, <8 mg/kg. RR, risk ratio. CI, confidence interval.

### Dosing of PZA in single-drug preparation

Taiwan CDC recommended 1500 mg PZA for patients weighing ≥50 kg and 1000 mg for those weighing <50 kg. The alternative recommendation is to prescribe 25 (range 20–30) mg/kg body weight with 2000 mg as the maximal dose.

**Table 3:** Factors associated with inadequate dosing of isoniazid among patients treated with Rifater, Rifinah or isoniazid in single-drug preparation, 2007–2010, Taipei.

	Adequate	Too high		Too low	
	Number (%)	Relative RR	95% CI	Relative RR	95% CI
Weight (kg)					
≤39	19(13.3%)	1		1	
40–49	58(7.2%)	2.64	1.36–5.14	0.44	0.06–3.43
≥50	706(35.1%)	0.02	0.01–0.04	0.13	0.03–0.72
Preparations					
Isoniazid	406(68.8%)	1		1	
Rifater	5(0.3%)	12017.89	4215.91–34258.24	103.84	23.12–466.32
Rifinah150	25(19.1%)	16.93	7.62–37.60	4.91	0.94–25.54
Rifinah300	347(90.4%)	0.63	0.34–1.13	0.23	0.03–1.91

Adequate dosage (4–6 mg/kg) as the base for comparison. Too high, >6 mg/kg. Too low, <4 mg/kg. RR, risk ratio. CI, confidence interval.

### Dosing of EMB in single-drug preparation

Taiwan CDC recommended 800 mg EMB for patients weighing 40–55 kg, 1200 mg for patients weighing 56–75 kg, and 1600mg for patients weighting 76–90 kg. The alternative recommendation is to prescribe 15 (range 15–20) mg/kg body weight.

**Table 4:** Factors associated with inadequate dosing of pyrazinamide among patients treated with Rifater or pyrazinamide in single-drug preparation, 2007–2010, Taipei.

	Adequate	Too high		Too low	
	Number (%)	Relative RR	95% CI	Relative RR	95% CI
Sex					
Female	699(82.9%)	1		1	
Male	1275(69.6%)	1.00	0.61–1.63	1.98	1.54–2.54
Weight (kg)					
≤39	92(77.3%)	1		1	
40–49	635(88.1%)	1.09	0.55–2.15	0.13	0.06–0.29
≥50	1247(68.0%)	0.01	0.002–0.05	2.66	1.49–4.75
Preparations					
Pyrazinamide	620(75.2%)	1		1	
Rifater	1354(73.2%)	0.04	0.02–0.08	2.05	1.63–2.57

Adequate dosage (20–30 mg/kg) as the base for comparison. Too high, >30 mg/kg. Too low, <20 mg/kg. RR, risk ratio. CI, confidence interval.

### Internationally-recommend dosages in mg/kg body weight

Dosages prescribed were further compared with internationally-recommend dosages in mg/kg body weight: 5 (4–6) mg/kg for INH, 10 (8–12) mg/kg for RMP, 15 (15–20) mg/kg for EMB and 25(20–30) mg/kg for PZA.

**Figure 8:**
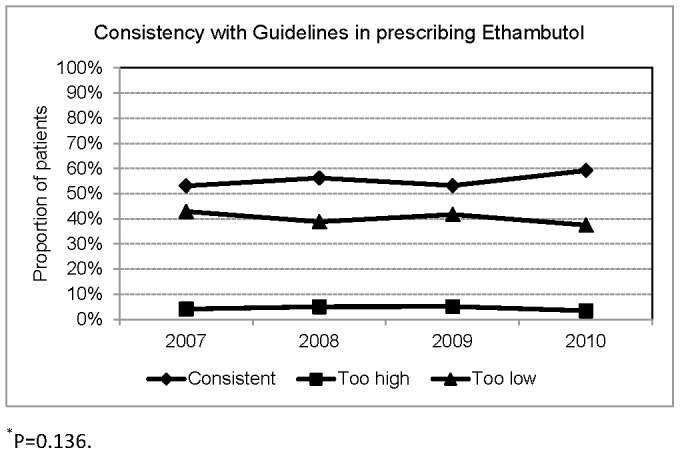
Consistency with Taiwan Guidelines in prescribing Ethambutol.

### Data entry and analysis

To ensure accuracy of data entry, the complete data set was double entered separately by 2 team members, and validated using EpiData Entry 3.1 (EpiData Association, Odense, Denmark). Discrepant records were checked and corrected against the original data on the questionnaires. STATA Version 12 (StataCorp LP, College Station, Texas, USA) was used for statistical analysis. Categorical variables were analysed using Pearson's χ2 test. *P*<0.05 was considered statistically significant. Relevant determinants were entered into multinomial logistic regression models in which the outcome variable had three categories: adequate dosage, lower-than-recommended dosage and higher-than-recommended dosage. Determinants remaining significant in the multinomial logistic regression models were retained and a final fitted model was determined by backward elimination using the likelihood ratio test.

**Table 5:** Factors associated with inadequate dosing of ethambutol, 2007–2010, Taipei.

	Adequate	Too high		Too low	
	Number (%)	Relative RR	95% CI	Relative RR	95% CI
Sex					
Female	576(64.7%)	1		1	
Male	764(39.2%)	1.90s	1,24–2.92	1.79	1.46–2.21
Weight (kg)	56(40.6%)				
≤39	699(90.9%)	1		1	
40–49	585(30.3%)	0.02	0.01–0.04	0.30	0.15–0.58
≥50	56(40.6%)	0.07	0.04–0.11	7.88	4.25–14.60

Adequate dosage (15–20 mg/kg) as the base for comparison. Too high, >20 mg/kg. Too low, <15 mg/kg. RR, risk ratio. CI, confidence interval.

### Ethics

The study was approved by the Taiwan CDC review board and was funded by the Taiwan CDC. The study reviewed routine surveillance data recorded on national TB registry and TB case management files and did not involve any human participation. Therefore, the project was not considered to require review by an ethics committee and informed consent was waived.

## Results

### Basal characteristics of the population

A total of 6790 TB cases were notified to Taipei health authorities and Taiwan CDC in 2007–2010. (Figure 1) Of the 3316 culture positive cases enrolled in this study, 3131 (94.4%) were treated with anti-TB drugs and 185 (5.6%) were not. Of the 3131 patients who were treated, 3020 (96.5%) had recorded pre-treatment body weight and 111(3.6%) had not. The proportion of patients who had no recorded pretreatment body weight was 12.6% in 2007, which decreased to 0.5% in 2008, 0.6% in 2009 and zero in 2010 (p<0.001).Of the 3020 patients with recorded pretreatment body weight, 2990 (99.0%) were adults. (Table 1).

### Dosage appropriateness and consistency with guidelines

Of the 2990 patients, 2627(87.9%) were prescribed HRZE, 209(7.0%) HRE, 89 (3.0%) HRZ, and 65 (2.2%) other regimens. Of the 2990 patients, 2917(97.6%) were treated with RMP containing regimens, in which 1849(63.4%) were treated with a 3-drug FDC, 514(17.6%) a 2-drug FDC, and 553(19.0%) RMP.

Among the 1849 patients treated with a 3-drug FDC (Rifater), the dosage was consistent with Taiwan guidelines in 1622(87.7%), too high in 162(8.8%), too low in 65(3.5%). Figure 2 shows that the proportion of patients with consistent dosage of Rifater was 83.7% in 2007, which increased to 91.4% in 2010 (P<0.001).

Of the 514 patients treated with 2-drug FDCs, 125 were treated with RFN150, 384 with RFN300, and 5 patients with both RFN150 and RFN300. Among the 125 patients treated with RFN150, the dosage was consistent in 91(72.8%) patients, too high in 15(12.0%), and too low in 19(15.2%). The proportion of patients treated with too low a dosage of RFN150 was 27.8% in 2007, which decreased to 12.5% in 2010 (p = 0.138). Among the 384 patients treated with RFN300, the dosages were consistent with Taiwan guidelines in 347(90.4%), too high in 36(9.4%), and too low in 1(0.3%). The proportion of patients treated with too low a dosage of RFN300 was 1.4% in 2007, which decreased to 0 in 2010 (p = 0.455).

Due to unbalance ratios of H:R:Z in Rifater and of H:R in RFN150, the dosage of INH in mg/kg body weight was too high in 99.4% of patients treated with Rifater (Figure 3) and in 81.6% of patients treated with RFN150 (Figure 4).

Among the 553 patients treated with RMP, the dosage was consistent with Taiwan guidelines in 473(85.5%), too high in 51(9.2%), too low in 29(5.2%). The proportion of patients treated with too low a dosage of RMP was 10.5% in 2007, which decreased to 0.9% in 2010 (P = 0.007).(Figure 5) Applying 10 mg/kg (range 8–12) as the recommended RMP dose, the dosage was adequate in 83.0%, too high in 11.7%, and too low in 5.2%.

A total of 590 patients were treated with INH. Applying the Taiwan guidelines' recommendation of 300 mg of INH for most patients and 200 mg for those weighing ≤40 kg, the dosage of INH was consistent in 562(95.3%), too high in 20(3.4%), and too low in 8(1.4%).(Figure 6) Applying 5 mg/kg (range 4–6) of INH as the recommended dosage, the dosage was adequate in 406(68.8%) patients, too high in 177(30.0%), too low in 7(1.2%).

A total of 825 patients were treated with PZA. Applying the Taiwan guidelines' recommendation, the dosage of PZA was adequate in 650(78.8%), too high in 71(8.6%), too low in 104(12.6%). The proportion of patients receiving too low a dosage of PZA was 19.8% in 2007, which decreased to 12.4% in 2010 (P = 0.001). (Figure 7) Applying 25 mg/kg (range 20–30) of PZA as the recommended dosage, the dosage was adequate in 75.2%, too high in 9.7%, too low in 15.2%.

Overall, among 2917patients treated with either Rifater, or Rifinah, or RMP in single-drug preparation, the dosage of RMP was adequate (8–12 mg/kg) in 2571(88.1%) patients, too high in 282(9.7%), too low in 64(2.2%). In multinomial logistic regression models, factors significantly associated with adequate dosage of RMP were body weight and preparations of RMP. (Table 2) Patients treated with Rifater (rrr 0.2, 95% confidence interval (CI) 0.1–0.4) and Rifinah300 (rrr 0.04, 95% CI 0.01–0.27) were less likely to have lower-than-recommended dose of RMP as compared with patients treated with RMP in single-drug preparation.

Overall, among 2954 patients treated with either Rifater, or Rifinah, or INH in single-drug preparation, the dosage of INH was adequate (4–6 mg/kg) in 783(26.5%), too high in 2155(73.0%), too low in 16(0.5%). In multinomial logistic regression models, factors significantly associated with adequate dosage of INH were body weight and preparations of INH. (Table 3) Patients treated with Rifater (rrr 12017.9, 95% CI 4215.9 – 34258.2) and Rifinah150 (rrr 16.9, 95% CI 7.6 – 37.6) were more likely to have higher-than-recommended dose of INH as compared with patients treated with INH in single-drug preparation.

Overall, among 2674 patients treated with either Rifater or PZA, the dosage of PZA is adequate (20–30 mg/kg) in 1974(73.8%) patients, too high in 88(3.3%), too low in 612(22.9%). In multinomial logistic regression models, factors significantly associated with adequate dosage of PZA were sex, body weight and preparations of PZA. (Table 4) Male (rrr 2.0, 95% CI 1.5–2.5), patients weighing ≥50 kg (rrr 2.7, 95% CI 1.5–4.8), and patients treated with Rifater (rrr 2.1, 95% CI 1.6–2.6) were more likely to receive lower-than-recommended dose of PZA as compared with other groups.

A total of 2841 patients were treated with EMB. The dosage of EMB was consistent with Taiwan guidelines for 1575(55.4%), too high for 125(4.4%), too low for 1141(40.2%). (Figure 8) Applying 15 mg/kg (range 15–20) of EMB as the recommended dosage, the dosage was adequate for 1340(47.2%) patients, too high for 150(5.3%), too low for 1351(47.6%). In multinomial logistic regression models, factors significantly associated with adequate dosage of EMB were body weight and sex. (Table 5) Patients weighing ≥50 kg were more likely to receive lower-than-recommended dose of EMB (rrr 7.9, 95% CI 4.3–14.6).

### Comparison with the pre-intervention period

In summary, the proportion of patients with recorded pre-treatment body weight was 64.5% in 2003, which increased to 96.5% in 2007–2010 (p<0.001). The proportion of patients treated with consistent dosing of Rifater increased from 73.9% in 2003 to 87.7% in 2007–2010 (p<0.001), and that for Rifinah from 76.0% to 86.1% (p = 0.024), for RMP from 62.8% to 85.5% (p<0.001), and for INH from 87.8% to 95.3% (p<0.001).

## Discussion

The study showed that it was possible to strength prescribing practice in the treatment of TB among general practitioners. The proportion of TB patients notified in Taipei City who did not have recorded pretreatment body reduced to none in 2010. The proportion of patients treated with consistent dosages of anti-TB drugs increased substantially and the proportion of patients treated with lower-than-recommended dosage reduced drastically. This was remarkable as it was usually difficult to involve general practitioners in TB services [11]–[14]. Improvement in prescribing practice was observed prior to routinely capturing pre-treatment body weight, likely due to training of general practitioners and certification as TB services providers. Our experience demonstrated that it was feasible to train public health nurses in assessing adequacy in dosing anti-TB drugs. However, public health nurses were not able to directly advise general practitioners on prescription of anti-TB drugs, for which TB expert committee was essential. Taiwan CDC launched an ambitious plan in 2006 to reduce TB incidence by half in 10 years [15] and has been determined in tackling inadequate dosing of anti-TB drugs as it was not productive to hire outreach workers to perform directly-observed therapy of inadequate anti-TB regimens. Collaboration with the NHI program in reducing reimbursement for inadequate dosing was a creative approach, which likely played an important role in ensuring adequacy in prescribing anti-TB drugs. However, we were not able to attribute impact of each intervention independently.

Prescribing practices of PZA and EMB were less satisfactory, especially EMB, partly because prescribing practicing of PZA and EMB was not analyzed in 2003, partly because the role of EMB in the treatment of TB was not as prominent as RMP and INH. Many practitioners in Taiwan were not used to prescribe more than 800 mg EMB, due to the concern of adverse drug effect of EMB. The impact of inadequate dosing of EMB related to outcome of treatment and generation of drug resistance need further evaluation.

Although prescribing practice has been more consistent with Taiwan guidelines, the preparation of Rifater available in Taiwan had unbalance ratios of INH, RMP and PZA (1∶1.5∶3.1), and RFN150 had an unbalance ratio of INH and RMP (1∶1.5). As a result, dosing of RMP in an adequate range resulted in higher-than-recommended doses of INH in patients treated with Rifater and RFN150, and lower-than-recommended doses of PZA in patients treated with Rifater. This was not the case of RFN300 as the ratio of INH and RMP in each tablet of RFN300 was 1∶2, which is consistent with the ratio of recommended dosages of INH and RMP in mg/kg body weight. To avoid over-dosing of INH and under-dosing of PZA, the preparations of FDCs must be modified [3].

Body weight was another important factor that was significantly associated with inadequate dosing of anti-TB drugs [16]. Patients who had relatively lower body weight were prone to receive higher-than-recommended dosage and those who had relatively higher body weight lower-than-recommended dosage. The exception is INH. The majority of patients were treated with >6mg/kg INH, due to inadequate preparation of FDCs and the recommendation of 300mg INH for the majority. It has been reported that a substantial proportion of patients who had unsatisfactory response to treatment had suboptimal serum concentration of RMP [17], and that higher than currently recommended dose of RMP might be useful [18], [19]. Therefore, it is essential not to prescribe lower-than-recommended dosage of RMP. Recently WHO [20] published a Rapid Advise document with new dosage recommendations for the treatment of tuberculosis in children. Adequate dosing of anti-TB drugs in children also needs to be ensured.

In conclusion, prescribing practice in the treatment of TB in Taipei City has remarkably improved after health authorities implemented a series of interventions. To have further improvement on dosing of anti-TB drugs, inadequate preparation of FDCs needs to be addressed and prescribing practice of EMB needs additional attention. Other settings where prescribing practice of anti-TB drugs have not yet been assessed should be encouraged to conduct similar clinic audits.

## References

[1] Lo H-Y, Yang S-L, Chou P, Chuang J-H, Chiang C-Y (2011) Completeness and timeliness of tuberculosis notification in Taiwan. BMC Public Health 11(1): 915. 22151346 10.1186/1471-2458-11-915 PMC3260335

[2] International Union against Tuberculosis and Lung Disease (2010) Management of tuberculosis. A guide to the essentials of good practice. (Sixth edition). Paris.

[3] World Health Organization (2010) Treatment of tuberculosis: guidelines for national programmes. Fourth edition. World Health Organization Document WHO/HTM/TB/2009 420: 1–147.

[4] Chiang C-Y, Bai K-J, Lee C-N, Enarson DA, Suo J, et al (2010) Inconsistent dosing of anti-tuberculosis drugs in Taipei, Taiwan. Int J Tuberc Lung Dis 14: 878–883. 20550772

[5] Diop AH, Gakiria G, Pande SB, Malla P, Rieder HL (2002) Dosages of anti-tuberculosis medications in the national tuberculosis programs of Kenya, Nepal, and Senegal. Int J Tuberc Lung Dis 6: 215–221. 11934139

[6] Harries AD, Gausi F, Salaniponi FM (2004) Prescriptions and dosages of anti-tuberculosis drugs in the National Tuberculosis Control Programme of Malawi. Int J Tuberc Lung Dis 8: 724–729. 15182142

[7] Norval PY (2010) Review of prescribing practices: an essential tool for measuring the quality of tuberculosis services. Int J Tuberc Lung Dis 14: 795. 20550760

[8] Li Y-P, Chan P-C, Wang K-F, Yang C-H, Kuo H-S (2009) Introduction of reducing reimbursement from NHI to improve the inadequate regimen for TB control. Int J Tuberc Lung Dis 13 (suppl): s107.

[9] Chan P-C, Hsu C-B, Wang K-F, Lin H-C, Hung M-N, et al. (2009) The impact of national endorsement of standardized regimens on TB care. Int J Tuberc Lung Dis 13 (suppl): s207.

[10] Taiwan Centers for Disease Control, Department of Health (2008) Taiwan guideline for tuberculosis diagnosis and treatment. 3rd ^ed^. Taipei.

[11] Uplekar M, Pathania V, Raviglione M (2001) Private practitioners and public health: weak links in tuberculosis control. Lancet 358: 912–916. 11567729 10.1016/S0140-6736(01)06076-7

[12] Udwadia ZF, Pinto LM, Uplekar MW (2010) Tuberculosis management by private practitioners in Mumbai, India: has anything changed in two decades? PLoS One 5(8): e12023 doi:10.1371/journal.pone.0012023. 20711502 10.1371/journal.pone.0012023 PMC2918510

[13] Lönnroth K, Uplekar M, Blanc L (2006) Hard gains through soft contracts: productive engagement of private providers in tuberculosis control. Bull World Health Organ 84: 876–883. 17143461 10.2471/blt.06.029983 PMC2627543

[14] Chiang C-Y, Trébucq A, Billo N, Khortwong P, Elmoghazy E, et al (2007) A survey of TB services in hospitals in seven large cities in Asia and North Africa. Int J Tuberc Lung Dis 11: 739–746. 17609048

[15] Centers for Disease Control. Department of Health. Republic of China. (2011) Mobilization of all citizens to reduce tuberculosis incidence by half in 10 years: Progress report. Taipei.

[16] Hoa NB, Lauritsen JM, Rieder HL (2012) Adequacy of anti-tuberculosis drug prescriptions in Viet Nam. Public Health Action 2: 5–9. 26392937 10.5588/pha.11.0015 PMC4536556

[17] Kimerling ME, Phillips P, Patterson P, Hall M, Robinson CA, et al (1998) Low serum antimycobacterial drug levels in non-HIV-infected tuberculosis patients. Chest 1998 113: 1178–1183. 10.1378/chest.113.5.1178 9596291

[18] Steingart KR, Jotblad S, Robsky K, Deck D, Hopewell PC, et al (2011) Higher-dose rifampin for the treatment of pulmonary tuberculosis: a systematic review. Int J Tuberc Lung Dis 15: 305–316. 21333096

[19] Ruslami R, Nijland HMJ, Alisjahbana B, Parwati I, van Crevel R, et al (2007) Pharmacokinetics and tolerability of a higher rifampin dose versus the standard dose in poulmonary tuberculosis patients. Antimicrob Agents Chemother 51: 2546–2551. 17452486 10.1128/AAC.01550-06 PMC1913243

[20] World Health Organization (2010) Rapid Advice: Treatment of tuberculosis in children. 26269860

