# Correction: Improved Consistency in Dosing Anti-Tuberculosis Drugs in Taipei, Taiwan

**DOI:** 10.1371/annotation/67e66370-247e-4406-b7a0-f0696d04e87e

**Published:** 2012-09-04

**Authors:** Chen-Yuan Chiang, Ming-Chih Yu, Hsiu-Chen Shih, Muh-Yong Yen, Yu-Ling Hsu, Shiang-Lin Yang, Tao-Ping Lin, Kuan-Jen Bai

Due to an error introduced during the production process, the Funding statement is incorrect. The correct Funding statement is: This work was funded by Centers for Disease Control, Department of Health, Taiwan, Republic of China. The funders had no role in study design, data collection and analysis, decision to publish or preparation of the manuscript. 

